# Fog computing: a platform for big-data marketing analytics

**DOI:** 10.3389/frai.2023.1242574

**Published:** 2023-10-04

**Authors:** Jacob Hornik, Matti Rachamim, Sergei Graguer

**Affiliations:** ^1^Coller School of Management, Tel-Aviv University, Tel Aviv, Israel; ^2^School of Business Administration, Bar-Ilan University, Ramat Gan, Israel; ^3^Ashkelon Academic College, Ashkelon, Israel

**Keywords:** fog computing, internet of things (IoT), cloud computing, edge computing, smart marketing

## Abstract

Marketing science embraces a wider variety of data types and measurement tools necessary for strategy, research, and applied decision making. Managing the marketing data generated by internet of things (IoT) sensors and actuators is one of the biggest challenges faced by marketing managers when deploying an IoT system. This short note shows how traditional cloud-based IoT systems are challenged by the large scale, heterogeneity, and high latency witnessed in some cloud ecosystems. It introduces researchers to one recent breakthrough, fog computing, an emerging concept that decentralizes applications, strategies, and data analytics into the network itself using a distributed and federated computing model. It transforms centralized cloud to distributed fog by bringing storage and computation closer to the user end. Fog computing is considered a novel marketplace phenomenon which can support AI and management strategies, especially for the design of “smart marketing”.

## 1. Introduction

“*Fog computing is considered a formidable next-generation complement to cloud computing*” (Sofla et al., 2022).

In recent years, with the technological advances in processors, memory and communications, a plethora of things has been updated with computational capacity, shaping what is known as cloud-based internet of things (IoT) systems. Moving into cloud computing represented a major shift because it replaced on-premises offerings requiring large, up-front payments with hosted computing resources made available on-demand on a pay-per-use pricing scheme (Taylor et al., 2020; Nezami et al., 2022). However, traditional cloud-based (IoT) systems are challenged by the large scale, heterogeneity, and high latency witnessed in some cloud ecosystems. Indeed, ubiquitous deployment of smart, interconnected devices is estimated to reach 58.2 billion units by 2025 (Costa et al., 2022). This exponential increase is fueled by the proliferation of mobile devices (e.g., mobile phones and tablets), with smart sensors serving different vertical markets, such as autonomous transportation, industrial controls, smart cities, smart homes, wireless sensors and actuators networks wearables, smart power grids, and smart management (Tomar et al., 2023). New concepts and technologies are needed to manage this growing fleet of IoT services creating massive amounts of “big-data.” Currently big-data is becoming a critical focus of research in marketing (e.g., Liu et al., 2016; Ma and Zhang, 2022; Li et al., 2023). However, relying on current technologies such as cloud computing is not efficient for addressing the requirements of big data management (Sofla et al., 2022). For this reason, a fog computing (FC) platform at the edge of the network was introduced to reduce the processing load from the cloud by delegating some simple and frequent tasks to the fog (Appendix Table 1). In November 2015, a coalition of academia and industry, including Microsoft, Intel, Cisco, ARM, Dell, and Princeton University, instituted the Open Fog Consortium to catalyze, publicize, and promote the implementation of FC. By 2018, fog computing had become a major platform for developing IoT infrastructure (Economist, 2018) involving academic institutions and governments. This platform has not yet been adopted in marketing, research and practice. Inspired by the recent successful applications of FC to many smart ecosystems, we propose some future application to marketing domains. To advance objectives, we first provide an overview of FC and related concepts relevant to marketing. Second, we elaborate on FC enabled marketing research (Tran-Dang and Kim, 2024).

## 2. Fog computing

Just as the natural phenomenon of fog is perceived when a cloud is close to the ground, the concept of FC was created to describe the computational paradigm that aims to bring the benefits of the cloud closer to end-devices. For this reason, it is considered a highly distributed computing paradigm, highly integrated with the cloud, but with the processing also being carried out at the edge of the network, enabling the execution of applications that had not been previously possible due to the high latency that existed in the communication between the devices and the cloud (Li et al., 2023; Tomar et al., 2023). FC presents the same functionalities that the cloud is offering to its users but with greater proximity and extent. The usual fog network rest on a cluster of heterogeneous interconnected sensors and devices used for computation, communication, and storage for latency-restricted IoT applications.

**Figure 1:**
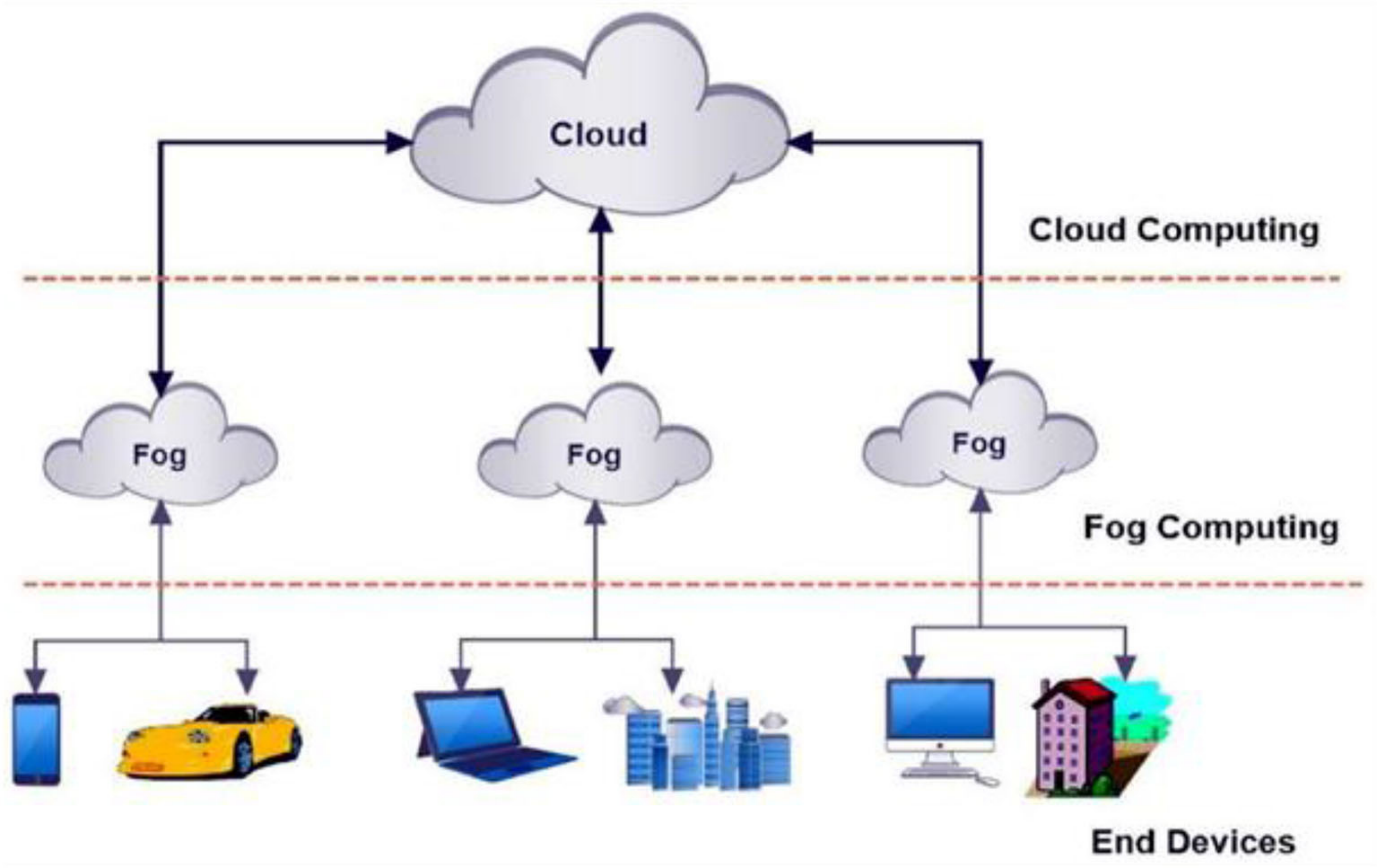
FC architecture (partially adopted from: Atlam et al., 2018).

FC architecture (Figure 1) is composed of highly dispersed heterogeneous devices with the intent of enabling deployment of IoT applications that requires storage, computation, and networking resources distributed at different geographical locations. FC is a layered model for enabling ubiquitous access to a shared continuum of scalable computing resources (Costa et al., 2022; Manzoor et al., 2022). The model facilitates the deployment of distributed, latency-aware applications and services, and consists of fog nodes (physical or virtual), residing between smart end-devices and centralized (cloud) services. FC minimizes the request-response time from/to supported applications, and provides, for the end-devices, local computing resources and, when needed, network connectivity to centralized services (Tran-Dang and Kim, 2024). Figure 1 depicts FC in the broader context of a cloud-based ecosystem serving smart end-devices. This essentially brings the advantages and power of the cloud closer to the place where the data is actually generated, benefiting the resource information algorithms. Thus, we envision FC as bridge between the cloud and the edge of the network that aims to facilitate the deployment of the newly emerging IoT marketing applications. The basic characteristics of FC are presented in Appendix 2. For marketing research, it is important to explain the core component of the fog computing architecture—the fog nodes.

### 2.1. Fog nodes

The FC layer consist of several FC nodes, which are responsible for developing transactions between different sections and the cloud environment. Fog nodes are either physical components (e.g., routers, switches, gateways, servers, etc.) or virtual components (e.g., virtual machines, virtualized switches, cloudlets, etc.) that are strongly connected to the smart end devices or access networks, and provide computing resources to these devices. Fog nodes provide some form of data management and communication services between a network's edge layer where end-devices reside, and the fog computing service or the centralized (cloud) computing resources, when needed (Li et al., 2023). Since FC is identified and defined as an extension of the traditional cloud-based computing model, the following deployment models important for marketing research are also supported:

Private fog node: a fog node that is provisioned for exclusive use by a single organization comprising multiple consumers (e.g., business units). It may be owned, managed, and operated by the organization, a third party, or some combination of them, and it may exist on or off premises.

Community fog node: a fog node that is provisioned for exclusive use by a specific community of consumers from organizations that have shared concerns (e.g., mission, security requirements, policy, and compliance considerations). It may be owned, managed, and operated by one or more of the organizations in the community, a third party, or some combination of them, and it may exist on or off premises.

Public fog node: a fog node that is provisioned for open use by the general public. It may be owned, managed, and operated by a business, academic, or government organization, or some combination of them. It exists on the premises of the fog provider.

Hybrid fog node: a complex fog node that is a composition of two or more distinct fog nodes (private, community, or public) that remain unique entities, but are bound together by standardized or proprietary technology that enables data and application portability.

## 3. SDN based fog computing

SDN has emerged as a software network architecture that allows the management of the complexity of FC environment and helps solving the IoT heterogeneity problem, enabling the creation of independent features and protocols of manufacturers, overcoming the problems related to the closed hardware and proprietary software (Kumhar and Bhatia, 2022). In order to solve the limitations of traditional network infrastructures, SDN was proposed. SDN-based FC is a type of computing architecture that combines SDN with FC. This approach enables the creation of a highly distributed network infrastructure that is capable of providing low-latency, high-bandwidth connectivity to a wide range of devices and applications. A significant amount of data is produced by the quickly evolving IoT and communication technologies. But traditional networks built with communication technologies are typically based on hardware, which restricts the network's potential to scale. SDN was developed as a standard to address scalability concerns and other constraints that limit the processing capability of traditional networks. It offers network scalability, flexibility, cheap costs, and low power consumption (Das and Inuwa, 2023).

**Figure 2:**
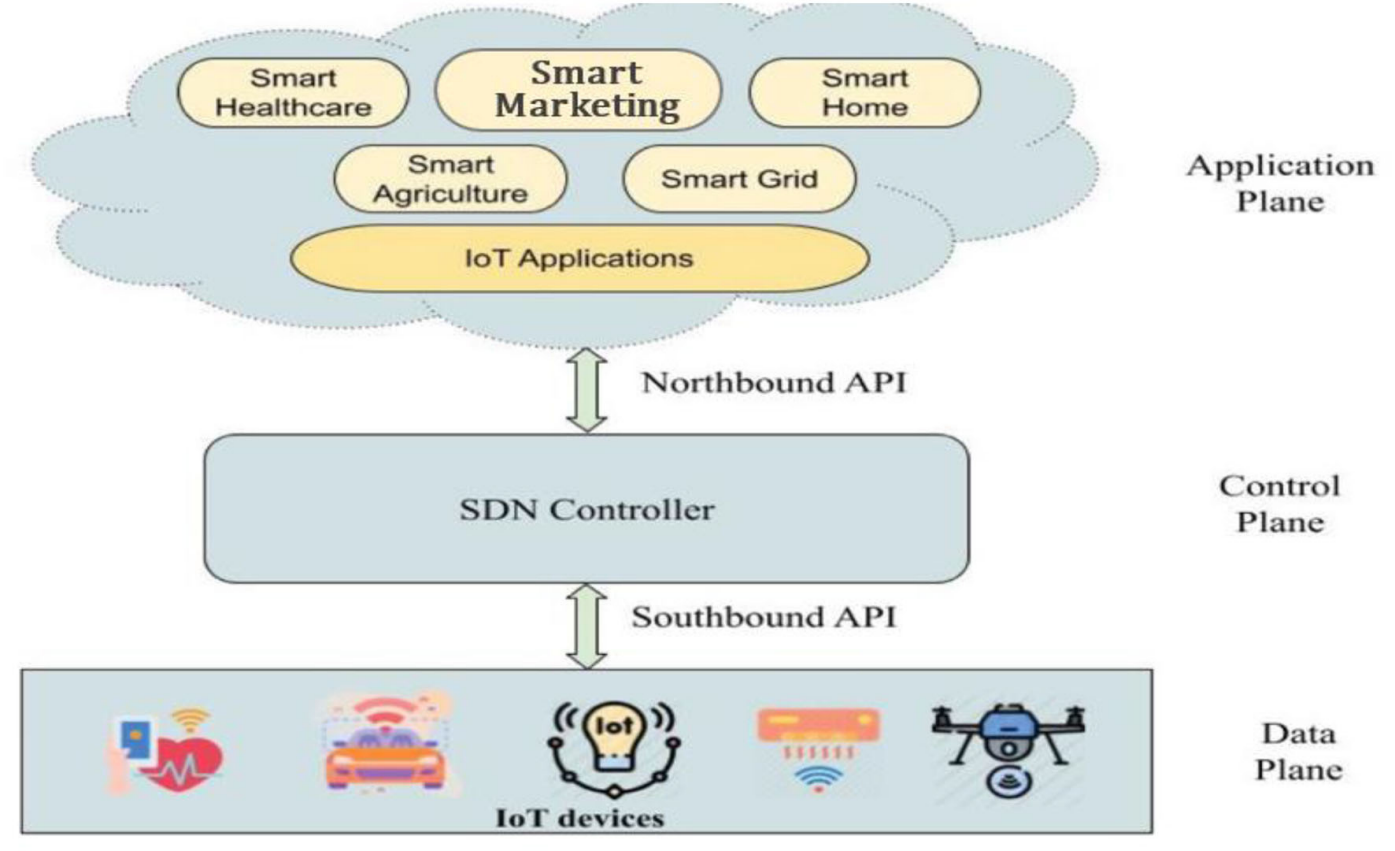
SDN based FC architecture (partially adopted from https://ideas.repec.org/a/aif/journl/v5y2021i6p117-130.html).

In traditional networks, the control plane and the data plane are located within the network elements, requiring a configuration on each device, using a low-level and often vendor-specific commands. SDN, unlike the traditional network, and, as shown in Figure 2, it separates the control plane from the data plane, having a centralized control that provides an abstract overview of all the network topology. Thus, it is possible to optimize service management as well as support service requirement from a centralized user interface (UI), offering greater agility, programmability and the capability to implement network automation (Ahmad et al., 2020). Unlike traditional IP networks in which the control and data planes are tightly coupled and embedded in the same networking elements, SDN separates the control plane from the data plane. In SDN, control is migrated out of the network elements and into the separate, centralized controller. This control and data planes separation in SDN brings some advantages: (1) it can break the vertical integration, and (2) it can simplify policy enforcement and network (re)configuration and evolution. It is because that the network elements (i.e., switches and routers) become simple forwarding devices, and there is a logically centralized controller (Das and Inuwa, 2023).

Figure 2 depicts the three-plane SDN-based IoT architecture. The data plane consists of smart IoT devices that sense and generate the data. The control plane acts as a brain for the core network because it has a centralized view of the entire network. The SDN controller and the network devices communicate with each other through the southbound API. Application plane can consist of diverse FC applications such as smart marketing smart home, smart healthcare, intelligent transport, smart agriculture, smart grid, etc. The application and control planes communicate with each other through the northbound API. The FC has a fast-growing number of devices, and programmable models like SDN help to provide quality of service (Kumhar and Bhatia, 2022). It divides the network's control and forwarding operations, which are referred to as the control plane and data plane. In sum, this kind of software can change the manual operation of repetitive marketing systems and replaced it with performance oriented application software constructed for specific marketing research purposes.

## 4. FC applications

FC is constantly modified and the number of applications running in a fog platform is growing (Li et al., 2023; Tomar et al., 2023; Tran-Dang and Kim, 2024). The new structure and use cases of fog platform are driven by an innovative application design that requires additional platform characteristics which can only be delivered if the applications are deployed next to the end users, as in automotive, supply chain and healthcare systems (“Patient monitoring,” 2022, http://www.fogguru.eu/tmp/OpenFog-Use-Cases.zip), to name a few. Modern marketing routinely captures “big-data” on large amounts of observations across subjects, brand SKUs, time periods, predictor variables, and store locations, thereby generating massive multi-faceted datasets. For example, Amazon and Aliexpress have choice data on millions of items selected, user ratings, and geodemographic profiles. Similar datasets were developed in retailing with potential use of RFIDs (radio frequency identifications), online auctions, product reviews, mobile marketing, social networking sites, internet commerce, and customer relationship. Choice modeling of such very large databases poses new computational issues, and the sophistication of academic researchers. Specifically, using customer feedback in the item design and manufacturing, so that the platform can provide user-based product development (Naik et al., 2008). The capacity to classify marketing data and make important judgments in the device's own context will assist in extracting essential information from the large volume of available marketing datasets. Thus, the marketing-monitoring system can be designed with the help of IoT and FC to minimize the latency in processing the massive marketing data. The data-sharing configurations and authentication are calculated automatically based on node verification and smart modules. The transmitted data is protected, preventing the risk of data tampering in the public cloud environment. Thus, FC is an important object of research among many disciplines (Appendix 3 lists major publications). Case in point, supply chain management.

### 4.1. FC in supply chain management

Gupta and Singh (2023) have elaborated how FC can be used in supply chain management. They claim that increased competition and customer expectations have negatively impacted supply chain performances, the environment, and operational costs. Risk to safety, quality, traceability, and transparency has increased many folds. Being an extension for cloud, FC provides supports to supply chain operations and strategies with efficient resource utilization, reduces risks, identification, bandwidth utilization, enhanced performances, tracking, energy consumption, and above all real-time monitoring. Otherwise, the heterogeneity of available platforms and services makes all these supports difficult. Innovative FC, IoT, and cloud computing infuse novel approaches to assimilate, store, and share transactional data through collaboration and interoperability among marketing stakeholders. FC supports real-time data processing with the help of time-sensitive applications. Gupta and Singh (2023) suggested an approach for improving supply chain performance based o FC, IoT technology, and cloud computing and mitigating latency issues. In addition to FC in supply chain management, FC can provide a range of exciting possibilities to facilitating and enhance marketing analytics. It is important to emphasize that the most significant contribution to marketing analytics is the ability of FC to integrate and synthesize even convoluted, unstructured data (numerical, semantic, emotional, and sensory) and immersive technologies to a unified and standardize interoperability algorithmic workable information. All to assist marketing decisions-makers and research capabilities in the different marketing ecosystems.

## 5. Research agenda

In addition to the obvious questions, we may ask if FC can embrace the variety of datasets and measurement devices necessary for some of the applied marketing decision making and choice research. Other questions concern how to fully realize the important developments and applications of the FC platform to marketing strategy, the types of marketing devices and services that can be moved into this platform, and the future of marketing research as a result of FC integration. Among the many interesting research questions are the following.

RQ1: Given that FC incorporates elements of the cognitive process, can it integrate emotional sources, such as emotionally intelligent machines, which are so important to marketing research? (Caruelle et al., 2022).
RQ2: What types of marketing devices and services can be moved into this platform?
RQ3: How can FC contribute to improving supply chain strategic performance and mitigating the latency issue?
RQ4: How can marketing utilize the FC architecture for maximizing customer satisfaction, market share and profitability?
RQ5: Are all security and privacy issues important to marketing solved by FC?
RQ6: Given that, detecting and selecting the relevant fog node in a high-mobility marketing environment is challenging (Costa et al., 2022), can FC fully coordinate among the many mobile fog nods?
RQ7: What are the offloading capabilities of FC when the aim of offloading is to outsource the load exchanges between fog nodes or between fog nodes and the cloud? (Sofla et al., 2022).
RQ8: How can a blockchain and FC collaborative design support marketing research and strategy? (Jain et al., 2021).

## 6. Conclusions

According Martin Key et al. (2020, p. 162) “Marketing scholars tend to ignore new technologies, and this is particularly true of new information technologies…” FC is an exciting novel system to network architecture that can help marketing to overcome the limitations imposed by traditional cloud networks. The many advantages of FC are clear, including reduced transmission costs and analysis for IoT, and better near-real-time response, which will have a significant influence on operation of distribution networks, supply chains, factories, cybersecurity services, organizations, and even the quality of human lives. Also, since its inception, FC has played a major role in investigating the quality of service (QoS). But, with the evolution of the IoT and AI services and technologies, which established a new “smart world” where everything is monitored automatically, providing only quality of service is no longer acceptable as it does not provide a satisfactory user experience. Indeed, quality of experience (QoE), which gratifies user experience and enhances user performance, becomes vital and FC is considered as a major technological breakthrough (Vambe, 2023; Tran-Dang and Kim, 2024).

To summarize, “The era of the cloud's total dominance is drawing to a close” (Economist, 2018). Thus, FC will continue to evolve and position itself as crucial enablers of autonomous networks, which will strongly impact the performance of emerging use cases, such as smart products, immersive supply chain, and autonomous smart retailing. There are many opportunities for marketing research and strategy. We hope that this short note will encourage managers to adopt this nascent platform to advance strategy, research and theory.

## Author contributions

MR contributed to the literature review and paper conceptualization. SG contributed to the writing–original draft, writing–review and editing, methodology, validation, and visualization. All authors contributed to the article and approved the submitted version.
