# Supplementary material for: Fog computing: a platform for big-data marketing analytics
Source: Front Artif Intell. 2023 Oct 4;6:1242574. doi: 10.3389/frai.2023.1242574 (PMC10582701; doi:10.3389/frai.2023.1242574)
Supplement: Supplementary file 1 [file Table_1.DOCX]

**FOG COMPUTING: A PLATFORM FOR BIG-DATA MARKETING ANALYTICS**

WEB APPENDIXES

Web Appendix 1, Table 1: **Summary comparison between Cloud and Fog computing**

| ***Requirement*** | **Cloud Computing Fog Computing** |
| --- | --- |
| *Latency & Jitter* | High/medium Low |
| *Location of service* | Within Internet Network Edge |
| *Distance between data sources/consumers* | Multiple Hops Single Hop |
| *Location awareness* | No Yes |
| *Geo-distribution* | Centralized (Data Center) Distributed |
| *Number of nodes* | Large Larger |
| *Support for mobility* | No Yes |
| *Data analytics* | Data at Rest Data in Motion |
| *Connectivity* | Wire-line Wireless |

Web Appendix 2: **Fog computing offers the following benefits:**  (a) *Reduced latency*: Processing devices if placed closer to the devices reduces the latency as physical distance is reduced and response time will be much lesser as compared to when placed on data center. (b) *Energy efficiency*: Instead of sensors actually working all the time, gateways can act as communication proxies which can handle any request when sensors are on sleep mode and can be processed when sensors wake up. This is how energy efficiency can be improved within sensor devices. (c) *Bandwidth*: Instead of sending entire data to data center, large chunks of raw data can be processed at fog nodes, so as to reduce the volume of data sent to data center. (d) *Privacy*: Propagation of data can be reduced by means of fog computing. Sensitive data can be analyzed at local gateway rather than at a data center that is not in control of the user, so as to ensure privacy of user data.

**Advantages:**

Contextual location awareness, and low latency: Fog computing offers the lowest-possible latency due to the fog nodes’ awareness of their logical location in the context of the entire systems and of the latency costs for communicating with other nodes. The origins of fog computing can be traced to early proposals supporting endpoints with rich services at the edge of the network, including applications with low latency requirements. Because fog nodes are often co-located with the smart end-devices, analysis and response to data generated by these devices is much quicker than from a centralized cloud service or data center. Geographical distribution: In sharp contrast to the more centralized cloud, the services and applications targeted by the fog computing demand widely, but geographically-identifiable, distributed deployments. For instance, the fog computing will play an active role in delivering high quality streaming services to moving vehicles, through proxies and access points geographically positioned along highways and tracks. Heterogeneity: Fog computing supports collection and processing of data of different form factors acquired through multiple types of network communication capabilities. Interoperability and federation: Seamless support of certain services (real-time streaming services is a good example) requires the cooperation of different providers. Hence, fog computing components must be able to interoperate, and services must be federated across domains. Real-time interactions: Fog computing applications involve real-time interactions rather than batch processing. Scalability and agility of federated, fog-node clusters: Fog computing is adaptive in nature, at cluster or cluster-of-clusters level, supporting elastic compute, resource pooling, data-load changes, and network condition variations, to list a few of the supported adaptive functions. Predominance of wireless access: Although fog computing is used in wired environments, the large scale of wireless sensors in IoT demand distributed analytics and compute. For this reason, fog computing is very well suited to wireless IoT access networks. Support for mobility: It is essential for many fog computing applications to communicate directly with mobile devices, and therefore support mobility techniques, such as the Locator/ID Separation Protocol (LISP)10, that decouple host identity from location identity, and require a distributed directory system.

Web Appendix 3: **Fog nodes characteristics**

1. Computing power: Fog nodes possess computational capabilities that allow them to execute various tasks and applications. They can process data, run software, and perform analytics at the edge of the network.
2. Storage capacity: Fog nodes typically have local storage to store and manage data. This local storage can be used to cache frequently accessed data, store intermediate results, or even perform local data persistence.
3. Networking capabilities: Fog nodes are equipped with network interfaces that enable communication with other nodes, devices, or the cloud. They can transmit and receive data, interact with other nodes in the fog computing infrastructure, and connect to the wider network.
4. Data filtering and processing: Fog nodes can filter, preprocess, and aggregate data locally before transmitting it to the cloud or other nodes. This reduces the amount of data that needs to be sent over the network and improves overall bandwidth efficiency.
5. Real-Time decision-making: Fog nodes enable real-time decision-making by processing data locally and generating immediate responses. This is particularly useful for time-sensitive marketing applications where low latency is crucial.
6. Scalability and heterogeneity: Fog computing environments can consist of a large number of diverse fog nodes. These nodes can vary in terms of their capabilities, processing power, and storage capacity. FC architectures are designed to accommodate heterogeneous resources and scale according to the requirements of the system.

Private fog node: a fog node that is provisioned for use by only a single organization comprising multiple users. It may be managed and operated by the organization users, a third party user, or some combination of them, and it may be located on or off the property. Community fog node: a fog node that is provisioned for use only by a specific community of users from organizations that have shared missions. It may be owned, and operated by one or more of the organizations in the locality, a third party, or some combination of them, and it may exist on or off the property. Public fog node: a fog node that is established for open use by the general public. It may be managed, and operated by a company, academic, or public organization, or some combination of them. It exists on the property of the fog provider. Hybrid fog node: a compound fog node that is a combination of two or more different fog nodes (private, or public, community) that continue as special entities, but are connected by standardized or proprietary technology that provides data and application portability.

Web Appendix 3: **“Smart” Applications**

**Smart Cities**

Smart cities are urban areas that use advanced technology and data-driven solutions to enhance the quality of life for its residents and visitors. These cities use connected devices, sensors, and other innovative technologies to improve the efficiency of public services, reduce resource consumption, and enhance the overall sustainability of the city. Smart cities use data to inform decision-making processes and improve the delivery of services such as transportation, energy, and public safety. They also prioritize citizen engagement and participation, ensuring that residents have access to information and are involved in shaping the future of their city. The goal of a smart city is to create a more livable, equitable, and sustainable urban environment for everyone.

The key features of smart cities include:

1. Information and Communication Technology (ICT) Infrastructure: Smart cities rely on a robust digital infrastructure that enables the seamless flow of information and communication between various components of the urban ecosystem.
2. Internet of Things (IoT) Sensors: IoT sensors and devices are deployed throughout the city to collect real-time data on various parameters such as traffic flow, air quality, energy consumption, waste management, and more. These sensors help in monitoring and optimizing urban systems.
3. Data Analytics: The vast amount of data collected from sensors and other sources is processed and analyzed to gain insights and make informed decisions. Data analytics enables city administrators to identify patterns, trends, and areas for improvement.
4. Sustainable Practices: Smart cities emphasize sustainability by incorporating renewable energy sources, implementing energy-efficient technologies, optimizing transportation systems, promoting recycling and waste management, and adopting eco-friendly practices.
5. Smart Mobility: Smart cities focus on intelligent transportation systems, which may include real-time traffic management, smart parking, electric vehicle infrastructure, bike-sharing programs, and public transportation systems integrated with digital platforms for efficient commuting.
6. Efficient Resource Management: Smart cities employ smart grids to optimize energy distribution, reduce wastage, and manage demand more effectively. They also implement smart water management systems to conserve water resources and detect leaks promptly.
7. Citizen Engagement and Participation: Smart cities actively involve residents in decision-making processes through digital platforms, mobile applications, and public feedback mechanisms. Citizens can contribute to the development and improvement of urban services.
8. Safety and Security: Smart cities employ technologies like video surveillance, emergency response systems, and predictive analytics to enhance public safety and security. These systems enable faster emergency response, crime prevention, and early detection of potential risks.
9. Improved Services: Through digital platforms and mobile applications, smart cities offer convenient and efficient services such as online government services, smart parking systems, intelligent waste management, and smart lighting.

The ultimate goal of smart cities is to create a sustainable, efficient, and livable urban environment that enhances the well-being of residents and enables economic growth. The specific technologies and initiatives implemented in smart cities can vary depending on the needs and priorities of each location.

**Smart product**

A smart product, also known as a connected or intelligent product, refers to a physical object or device that has embedded sensors, connectivity capabilities, and advanced technologies that enable it to collect and transmit data, interact with users or other devices, and provide enhanced functionality and convenience.

Smart products are typically part of the Internet of Things (IoT) ecosystem, where devices are interconnected and can communicate with each other and with the internet. They often have features that enable them to be controlled, monitored, or accessed remotely through mobile applications or web interfaces.

Here are some common characteristics and examples of smart products:

1. Connectivity: Smart products are connected to the internet or other devices, allowing them to communicate, send and receive data, and access online services or platforms. This connectivity enables remote control, data sharing, and real-time interactions.
2. Sensors and data collection: Smart products are equipped with various sensors, such as temperature, motion, light, or proximity sensors, to gather information about their environment or usage. They can collect data, monitor conditions, and provide insights for analysis and decision-making.
3. Automation and intelligence: Smart products often incorporate artificial intelligence (AI) or machine learning capabilities to automate tasks, learn user preferences, and adapt to changing conditions. They can make decisions, adjust settings, and provide personalized experiences based on collected data and user interactions.
4. Enhanced functionality and convenience: Smart products aim to improve user experiences and offer added features and convenience. Examples include smart home devices like thermostats, lighting systems, and security cameras that can be controlled remotely, or wearable fitness trackers that monitor activity levels and provide personalized health data.
5. Interoperability: Smart products are designed to work together, integrating with other devices, platforms, or ecosystems. They can communicate and share data with compatible devices, allowing for seamless interactions and creating a cohesive smart environment.
6. Data privacy and security: As smart products collect and transmit data, ensuring data privacy and security is crucial. Manufacturers implement measures to protect user data and devices from unauthorized access or misuse.

Some examples of smart products include smart speakers (e.g., Amazon Echo or Google Home), smart thermostats (e.g., Nest Thermostat), smartwatches, smart appliances (e.g., refrigerators, washing machines), smart locks, and smart lighting systems.

Smart products are transforming various industries, including home automation, healthcare, transportation, energy management, and agriculture. They offer new opportunities for efficiency, convenience, and customization, as well as improved monitoring, control, and decision-making capabilities for users.

**Smart banking**

Smart banking, also known as digital banking or online banking, refers to the use of digital technologies and online platforms to deliver banking services and enable financial transactions. It encompasses a range of electronic channels and tools that allow customers to access their accounts, conduct banking activities, and manage their finances conveniently and securely.

Here are some key features and components of smart banking:

1. Online Banking Platforms: Smart banking provides customers with access to secure online banking platforms, typically through web portals or mobile applications. These platforms enable customers to view account balances, transaction history, and statements, as well as perform various banking tasks such as fund transfers, bill payments, and account management.
2. Mobile Banking: Smart banking leverages mobile devices and dedicated banking applications to provide on-the-go banking services. Mobile banking apps enable customers to access their accounts, make transactions, and receive notifications through their smartphones or tablets. They often include features like fingerprint or facial recognition for secure login and authentication.
3. Digital Payments: Smart banking facilitates digital payment options, allowing customers to make payments and transfer funds electronically. This includes person-to-person (P2P) payments, mobile wallet payments, online bill payments, and integration with payment platforms such as Apple Pay, Google Pay, or other digital wallets.
4. Automated Transactions and Services: Smart banking automates routine banking transactions and services, reducing the need for in-person visits to a bank branch. This includes features such as automatic recurring bill payments, scheduled transfers, account alerts, and the ability to set up standing instructions for regular transactions.
5. Personalized Financial Management: Smart banking platforms often offer tools and features to help customers manage their finances effectively. These may include budgeting tools, spending categorization, financial goal setting, and real-time insights into spending patterns. Personalized recommendations and financial planning guidance may also be provided based on customer data analysis.
6. Enhanced Security Measures: Smart banking places a strong emphasis on security to protect customer information and transactions. It employs advanced security technologies, such as encryption, secure authentication methods, and multi-factor authentication, to safeguard customer data and prevent unauthorized access.
7. Customer Support and Assistance: Smart banking platforms typically provide customer support and assistance through online chat, secure messaging, or phone support. Customers can seek help, ask questions, or resolve issues without needing to visit a physical bank branch.
8. Integration with Third-Party Services: Smart banking platforms often integrate with third-party financial services and fintech applications. This enables customers to access additional services such as personal finance management apps, investment platforms, or loan comparison tools within the banking ecosystem.

Smart banking offers customers convenience, accessibility, and control over their financial activities. It allows them to manage their accounts, conduct transactions, and access banking services anytime and anywhere using digital devices. Smart banking also promotes efficiency, reduces paperwork, and enhances the overall customer experience by providing a range of digital tools and services in the financial domain.

**Smart grid (Electrical power)**

A smart grid refers to an advanced electrical power distribution system that utilizes digital technologies, two-way communication, and intelligent devices to enhance the efficiency, reliability, and sustainability of electricity generation, distribution, and consumption. It modernizes the traditional electricity grid by incorporating information technology, automation, and real-time monitoring to optimize energy management and enable more flexible and responsive energy networks.

Here are key characteristics and components of a smart grid:

1. Advanced Metering Infrastructure (AMI): Smart grids deploy smart meters, which enable two-way communication between consumers and utility companies. Smart meters provide real-time information on energy consumption, enable remote meter reading, and support dynamic pricing programs, allowing consumers to make informed decisions about their energy usage.
2. Distribution Automation: Smart grids incorporate automation and monitoring systems to improve the operation and maintenance of the distribution network. Intelligent devices, such as sensors, switches, and reclosers, can detect faults, isolate problem areas, and restore power more efficiently. This reduces outage durations and improves overall reliability.
3. Integration of Renewable Energy: Smart grids facilitate the integration of renewable energy sources, such as solar and wind power, into the electricity grid. They can manage the intermittent nature of renewable generation, balance supply and demand, and enable the efficient distribution of clean energy to consumers.
4. Demand Response: Smart grids enable demand response programs, which allow utilities to adjust electricity consumption during peak demand periods. Through communication with smart devices and appliances, consumers can voluntarily reduce or shift their energy usage in response to price signals or grid conditions, helping to alleviate stress on the grid and promote energy conservation.
5. Energy Management Systems: Smart grids incorporate sophisticated energy management systems that analyze data from smart meters, sensors, and other sources to optimize energy distribution and consumption. These systems provide real-time information on energy flows, identify inefficiencies, and enable utilities and consumers to make data-driven decisions to improve energy efficiency.
6. Grid Resilience and Self-Healing: Smart grids are designed to be resilient and self-healing in the face of disruptions or natural disasters. They can detect and isolate faults, reroute power flows, and restore service quickly. This improves the reliability and robustness of the electrical grid, minimizing the impact of outages and improving system reliability.
7. Enhanced Customer Engagement: Smart grids empower consumers by providing them with access to real-time energy data, allowing them to monitor and manage their energy consumption more effectively. Consumers can make informed choices about energy usage, participate in demand response programs, and potentially save on their energy bills.

The implementation of smart grids offers numerous benefits, including improved energy efficiency, reduced greenhouse gas emissions, enhanced grid reliability, increased integration of renewable energy sources, and better alignment between supply and demand. It enables more efficient and sustainable energy management while providing consumers with greater control over their energy usage.

Web Appendix 4: **FC Selected Publications**

BOOKS

Abbas, A., Khan, S. U., & Zomaya, A. Y. (2020). *Fog Computing: Theory and Practice*. John Wiley & Sons.

Rehan, M. M., & Rehmani, M. H. (Eds.). (2020). *Blockchain-Enabled Fog and Edge Computing: Concepts, Architectures and Applications: Concepts, Architectures and Applications*. CRC Press.

Tanwar, S., & Tanwar. (2021). *Fog computing for Healthcare 4.0 environments*. Springer International Publishing.‏

Tomar, R. et al., (2022), *Fog Computing Concepts, Frameworks, and Applications*. Routledge publishing.

ARTICLES

AboDoma, N., Shaaban, E., & Mostafa, A. (2022). Adaptive time-bound access control for internet of things in fog computing architecture. *International Journal of Computers and Applications,* 44(8), 779-790.‏

Ahmadi Z, Haghi Kashani M, Nikravan M, Mahdipour E (2021) Fog-based healthcare systems: A systematic review. *Multimedia Tools and Applications*

Aiswarya, S., Ramesh, K., Sasikumar, S., Sheema, D., & Prabha, B. (2022). Internet of Health Things: A Fog computing Paradigm. *In 2022 6th International Conference on Trends in Electronics and Informatics (ICOEI)* (598-604). IEEE.‏

AL-Amodi, S., Patra, S. S., Bhattacharya, S., Mohanty, J. R., Kumar, V., & Barik, R. K. (2022). Meta-heuristic Algorithm for Energy-Efficient Task Scheduling in Fog Computing. In *Recent Trends in Electronics and Communication* ( 915-925). Springer, Singapore.‏

Aliyu, F., Sheltami, T., Deriche, M., & Nasser, N. (2022). Human immune-based intrusion detection and prevention system for fog computing. *Journal of Network and Systems Management,* 30(1), 1-27.‏

Alreshidi, E. J. (2022). Introducing Fog Computing (FC) Technology to Internet of Things (IoT) Cloud-Based Anti-Theft Vehicles Solutions. *International Journal of System Dynamics Applications* (IJSDA), 11(3), 1-21.‏

Alsadie, D. (2022). Task Scheduling in Fog Computing-Classification, Review, Challenges and Future Directions. *International Journal of Computer Science & Network Security*, 22(4), 89-100.‏

AlShathri, S. I., Chelloug, S. A., & Hassan, D. S. (2022). Parallel Meta-Heuristics for Solving Dynamic Offloading in Fog Computing. *Mathematics,* 10(8), 1258.‏

Alvi, A. N., Javed, M. A., Hasanat, M. H. A., Khan, M. B., Saudagar, A. K. J., Alkhathami, M., & Farooq, U. (2022). Intelligent Task Offloading in Fog Computing Based Vehicular Networks. *Applied Sciences*, 12(9), 4521.‏

Alzoubi, Y. I., Al-Ahmad, A., & Kahtan, H. (2022). Blockchain technology as a Fog computing security and privacy solution: An overview. *Computer Communications*, 182, 129-152.‏

Al-Zinati M, Alrashdan R, Al-Duwairi B, Aloqaily M (2021) A re-organizing biosurveillanceframework based on fog and mobile edge computing. *Multimed Tools Appl* 80:16805–16825.

Antonini, M., Vecchio, M., & Antonelli, F. (2019). Fog computing architectures: A reference for practitioners. *IEEE Internet of Things Magazine*, 2(3), 19-25.‏

Attiya, I., Abualigah, L., Elsadek, D., Chelloug, S. A., & Abd Elaziz, M. (2022). An Intelligent Chimp Optimizer for Scheduling of IoT Application Tasks in Fog Computing*. Mathematics,* 10(7), 1100.‏

Azizi, S., Shojafar, M., Abawajy, J., & Buyya, R. (2022). Deadline-aware and energy-efficient IoT task scheduling in fog computing systems: A semi-greedy approach. *Journal of network and computer applications*, 201, 103333.‏

Balasubramanian S, Meyyappan T (2020) Game theory based ofoad and migration-enabled smartgateway for cloud of things in fog computing. *Computing in engineering and technology.* Springer, Heidelberg, pp 253–266.

Bashir, H., Lee, S., & Kim, K. H. (2022). Resource allocation through logistic regression and multicriteria decision making method in IoT fog computing. *Transactions on Emerging Telecommunications Technologies*, 33(2), e3824.‏

Bilal K, Khalid O, Erbad A, Khan SU (2018) Potentials, trends, and prospects in edge technologies: fog, cloudlet, mobile edge, and micro data centers. *Comput Netw* 130:94–120

Bouachir O, Aloqaily M, Tseng L, Boukerche A (2020) Blockchain and fog computing for cyberphysical systems: the case of smart industry. *Computer* 53(9):36–45

Chen S, Zheng Y, Lu W, Varadarajan V, Wang K (2019) Energy-optimal dynamic computation offloading for industrial IoT in fog computing. *IEEE Trans Green Commun Netw* 4(2):566

Costa, B., Bachiega Jr, J., de Carvalho, L. R., & Araujo, A. P. (2022). Orchestration in fog computing: A comprehensive survey. *ACM Computing Surveys* (CSUR), 55(2), 1-34.‏

Elwy, F., Aburukba, R., & Al-Ali, A. R. (2022). Role of Fog Computing in Smart Spaces. In *2022 IEEE International Conference on Edge Computing and Communications* (EDGE) (69-76). IEEE.‏

Gasmi, Kaouther, et al. (2022), A survey on computation offloading and service placement in fog computing-based IoT. The Journal of Supercomputing 78.2, 1983-2014.‏

Ghobaei-Arani M, Souri A, Rahmanian AA (2019) Resource management approaches in fog computing: a comprehensive review. *J Grid Comput* 18:1–42.

Goudarzi, M., Palaniswami, M., & Buyya, R. (2022). Scheduling IoT Applications in Edge and Fog Computing Environments: A Taxonomy and Future Directions. *ACM Computing Surveys (CSUR)*.‏ in print.

Haghi Kashani M, Ahmadzadeh A, Mahdipour E (2022) Load balancing algorithms in fog computing: A systematic review*. IEEE Transactions on Services Computing*, Forthcoming.

Haghi Kashani M, Rahmani AM, Jafari Navimipour N (2020) Quality of service-aware approaches in fog computing. *Int J Commun Syst* 33:e4340.

Hallappanavar, V. L., & Birje, M. N. (2022). Prediction of quality of service of fog nodes for service recommendation in fog computing based on trustworthiness of users. *Journal of Reliable Intelligent Environments*, 8(2), 193-210.‏

Hamdi, A. M. A., Hussain, F. K., & Hussain, O. K. (2022). Task offloading in vehicular fog computing: State-of-the-art and open issues. *Future Generation Computer Systems*.‏in print.

Hewa, T., Braeken, A., Liyanage, M., & Ylianttila, M. (2022). Fog computing and blockchain-based security service architecture for 5G industrial IoT-enabled cloud manufacturing. *IEEE Transactions on Industrial Informatics*, 18(10), 7174-7185.‏

Hosseinioun, P., Kheirabadi, M., Kamel Tabbakh, S. R., & Ghaemi, R. (2022). aTask scheduling approaches in fog computing: A survey. *Transactions on Emerging Telecommunications Technologies*, 33(3), e3792.‏

Idrees, S. K., & Idrees, A. K. (2022). New fog computing enabled lossless EEG data compression scheme in IoT networks. *Journal of Ambient Intelligence and Humanized Computing*, 13(6), 3257-3270.‏

Jamil, B., Ijaz, H., Shojafar, M., Munir, K., & Buyya, R. (2022). Resource Allocation and Task Scheduling in Fog Computing and Internet of Everything Environments: A Taxonomy, Review, and Future Directions*. ACM Computing Surveys* (CSUR), in print.

Kamruzzaman, M. M., Yan, B., Sarker, M. N. I., Alruwaili, O., Wu, M., & Alrashdi, I. (2022). Blockchain and Fog Computing in IoT-Driven Healthcare Services for Smart Cities. *Journal of Healthcare Engineering,* in print.‏

Keshari, N., Singh, D., & Maurya, A. K. (2022). A survey on Vehicular Fog Computing: Current state-of-the-art and future directions. *Vehicular Communications*, 100512.‏

Khezr, S., Yassine, A., & Benlamri, R. (2022). Towards a secure and dependable IoT data monetization using blockchain and fog computing. *Cluster Computing*, 1-14.‏

Kumari A, Tanwar S, Tyagi S, Kumar N (2018) Fog computing for Healthcare 4.0 environment:opportunities and challenges*. Comput Electr Eng* 72:1–13.

Lata, M., & Kumar, V. (2022). Fog Computing Infrastructure for Smart City Applications. In *Recent Advancements in ICT Infrastructure and Applications* (119-133). Springer, Singapore.‏

Li, Y., Li, H., Xu, G., Xiang, T., & Lu, R. (2022). Practical Privacy-Preserving Federated Learning in Vehicular Fog Computing. *IEEE Transactions on Vehicular Technology*, 71(5), 4692-4705.‏

Liu Y, Yu FR, Li X, Ji H, Leung VC (2018) Distributed resource allocation and computation ofloading in fog and cloud networks with non-orthogonal multiple access. *IEEE Trans Veh Technol* 67(12):12137–12151.

Liu, C., Wang, J., Zhou, L., & Rezaeipanah, A. (2022). Solving the multi-objective problem of IoT service placement in fog computing using cuckoo search algorithm. *Neural Processing Letters,* 54(3), 1823-1854.‏

Mahmud, R., Pallewatta, S., Goudarzi, M., & Buyya, R. (2022). Ifogsim2: An extended ifogsim simulator for mobility, clustering, and microservice management in edge and fog computing environments. *Journal of Systems and Software,* 190, 111351.‏

Manzoor, A., Shah, M. A., Khattak, H. A., Din, I. U., & Khan, M. K. (2022). Multi‐tier authentication schemes for fog computing: Architecture, security perspective, and challenges. *International Journal of Communication Systems*, 35(12), e4033.‏

Mao, W., Akgul, O. U., Mehrabi, A., Cho, B., Xiao, Y., & Ylä-Jääski, A. (2022). Data-driven capacity planning for vehicular fog computing. *IEEE Internet of Things Journal*.‏ in print.

Mas, L., Vilaplana, J., Mateo, J., & Solsona, F. (2022). A queuing theory model for fog computing. *The Journal of Supercomputing*, 78(8), 11138-11155.‏

Ometov, A., Molua, O. L., Komarov, M., & Nurmi, J. (2022). A survey of security in cloud, edge, and fog computing. *Sensors*, 22(3), 927.‏

Rahimi M, Songhorabadi M, Kashani MH (2020) Fog-based smart homes: a systematic review. *JNetw Comput Appl* 153:102531

Rahman G, Chuah CW (2018) Fog computing, applications, security and challenges, review. *Int J Eng Technol* 7(3):1615–1621.

Rani, S., Kataria, A., & Chauhan, M. (2022). Fog computing in industry 4.0: Applications and challenges—A research roadmap. *Energy Conservation Solutions for Fog-Edge Computing Paradigms*, 173-190.‏

Rayes, A., & Salam, S. (2022). *Fog computing. In Internet of Things from hype to reality* (pp. 153-178). Springer, Cham.‏

Shakarami, A., et al.,(2022). Resource provisioning in edge/fog computing: A Comprehensive and Systematic Review. *Journal of Systems Architecture*, 122, 102362.‏

Sicari, S., Rizzardi, A., & Coen-Porisini, A. (2022). Insights into security and privacy towards fog computing evolution. *Computers & Security*, 102822.‏

Singh SP, Nayyar A, Kumar R, Sharma A (2018) Fog computing: from architecture to edge computing and big data processing*. J Supercomput* 75(4):2070–2105

Songhorabadi M, Rahimi M, Farid AMM, Kashani MH (2020) Fog computing approaches in smart cities: a state-of-the-art review. *Accessed https://arxiv.org/abs/2011.14732*

Wang X, Ning Z, Wang L (2018) Offloading in Internet of vehicles: a fog-enabled real-time traffic management system. *IEEE Trans Ind Inf* 14(10):4568–4578

Wang T, Zhou J, Liu A, Bhuiyan MZA, Wang G, Jia W (2018) Fog-based computing and storage offloading for data synchronization in IoT. *IEEE Internet Things J* 6:4272

Wu Q, Ge H, Liu H, Fan Q, Li Z, Wang Z (2019) A task offloading scheme in vehicular fog and cloud computing system. *IEEE Access* 8:1173.

Yadav, A. M., Tripathi, K. N., & Sharma, S. C. (2022). A bi-objective task scheduling approach in fog computing using hybrid fireworks algorithm. *The Journal of Supercomputing*, 78(3), 4236-4260.‏

Yadav, A. M., Tripathi, K. N., & Sharma, S. C. (2022). An enhanced multi-objective fireworks algorithm for task scheduling in fog computing environment. *Cluster Computing*, 25(2), 983-998.‏

Yang Y, Liu X, Guo W, Zheng X, Dong C, Liu Z (2020) Multimedia access control with secure provenance in fog-cloud computing networks. *Multimed Tools Appl* 79(15):10701–10716.

Yin, Z., Xu, F., Li, Y., Fan, C., Zhang, F., Han, G., & Bi, Y. (2022). A Multi-Objective Task Scheduling Strategy for Intelligent Production Line Based on Cloud-Fog Computing. *Sensors,* 22(4), 1555.‏

Yousefpour A, Ishigaki G, Gour R, Jue JP (2018) On reducing IoT service delay via fog offloading. *IEEE Internet Things J* 5(2):998–1010
